# New precision medicine avenues to the prevention of Alzheimer’s disease from insights into the structure and function of γ-secretases

**DOI:** 10.1038/s44318-024-00057-w

**Published:** 2024-02-23

**Authors:** Bart De Strooper, Eric Karran

**Affiliations:** 1 Dementia Research Institute, Institute of Neurology, University College London, at the Francis Crick Institute, London, NW1 AT UK; 2 Laboratory for the Research of Neurodegenerative Diseases, VIB Center for Brain & Disease Research, and Leuven Brain Institute, KU Leuven, Leuven, 3000 Belgium; 3 Cambridge Research Center, AbbVie, Inc., Cambridge, MA USA

**Keywords:** Presenilin, Gamma-secretase-modulator, γ-Secretase Allosteric Stabilizer, Alzheimer, Cancer, Cancer, Neuroscience, Pharmacology & Drug Discovery

## Abstract

Two phase-III clinical trials with anti-amyloid peptide antibodies have met their primary goal, i.e. slowing of Alzheimer’s disease (AD) progression. However, antibody therapy may not be the optimal therapeutic modality for AD prevention, as we will discuss in the context of the earlier small molecules described as “γ-secretase modulators” (GSM). We review here the structure, function, and pathobiology of γ-secretases, with a focus on how mutations in presenilin genes result in early-onset AD. Significant progress has been made in generating compounds that act in a manner opposite to pathogenic presenilin mutations: they stabilize the proteinase-substrate complex, thereby increasing the processivity of substrate cleavage and altering the size spectrum of Aβ peptides produced. We propose the term “γ-secretase allosteric stabilizers” (GSAS) to distinguish these compounds from the rather heterogenous class of GSM. The GSAS represent, in theory, a precision medicine approach to the prevention of amyloid deposition, as they specifically target a discrete aspect in a complex cell biological signalling mechanism that initiates the pathological processes leading to Alzheimer’s disease.

## Abstract (web summary)

This review explains how allosteric stabilizers of γ-secretase may prevent Alzheimer’s disease by specifically exerting opposite effects as pathogenic presenilin mutations.

## Graphical abstract

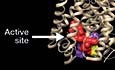

## Introduction

Alzheimer’s disease (AD) arguably represents one of the biggest unmet medical needs of our time. It is a complex disorder that evolves from a clinically silent biochemical phase during which amyloid plaques accumulate, to a cellular phase driven by neuroinflammation and Tau aggregation, ultimately causing synaptic dysfunction and cell death (De Strooper and Karran, 2016). The clinical phase of the disease starts decades after the initial biochemical phase and is characterized by progressive cognitive deterioration leading to dementia and death. Therapeutic strategies are likely to produce greater clinical benefit if they are administered as early as possible in the disease process, as recent successful trials with anti-amyloid antibodies (Sims et al, 2023; van Dyck et al, 2023) have demonstrated. Ideally, therapeutic intervention would prevent the first pathognomonic sign of AD—amyloid aggregation and the early cellular response to it—thus preventing irreversible damage to the brain.

**Figure 1:**
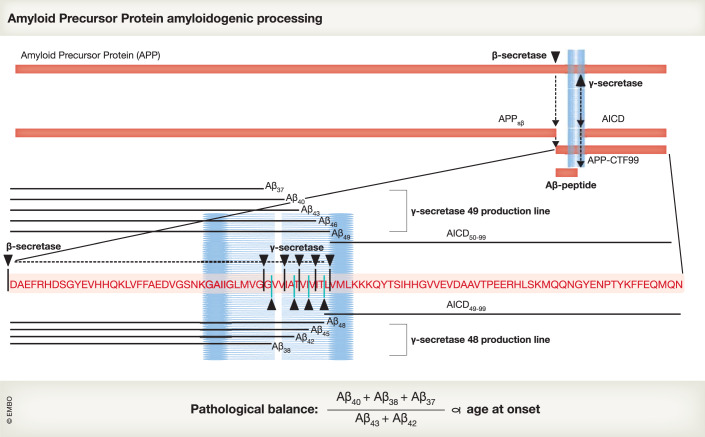
Processing of amyloid precursor protein to long and short amyloid peptides (Aβ). Amyloid precursor protein (APP) is a type I transmembrane protein, indicated at the top. The transmembrane domain (inserted in the membrane, blue) and the position of the β- and γ-secretase cleavage sites are indicated schematically. Beta-secretase is a type I transmembrane aspartyl-protease. We use in the text another name for β-secretase i.e., BACE1 (β-site amyloid precursor protein cleaving enzyme), because this name is preferred in the clinical trial literature. The β-secretase yields a soluble APP_sβ_ that is secreted in the medium, and a membrane-bound APP carboxyterminal fragment that is 99 amino acid-long (APP-CTF99) and remains membrane bound. The primary amino acid sequence of APP-CTF99 is displayed and the precise positions of the β-secretase and the consecutive γ-secretase cleavage sites are indicated. The combined actions of β- and γ-secretases generate Aβ peptides of different lengths, as indicated by the black lines above and below the primary amino acid sequence. The γ-secretase complex (Fig. 2), which is also an aspartyl-protease, cleaves APP-CTF99 first close to the intracellular site of the cell membrane to generate Aβ_49_ and AICD_50-99_ (49-production line), or Aβ_48_ and AICD_49-99_ (48-production line). The AICD is released in the cytoplasm, while the Aβ_48_ and Aβ_49_ remain associated with the γ-secretase complex. The Aβ peptides are further trimmed by consecutive cleavages by γ-secretase removing tri- or tetrapeptides at each step (Takami et al, 2009). Alzheimer’s disease-causing mutations in the presenilin subunit of the γ-secretase complex destabilize the interaction with the Aβ peptides increasing the chance of premature release of incompletely digested Aβ species. This results in relative shifts of long versus short Aβ peptides, as explained in the text. Under physiological conditions, the most abundant peptide generated in this process is Aβ_40_, and clinical mutations increase the release of Aβ_42_ and/or Aβ_43_. However, currently, only Aβ_37_, Aβ_38_, Aβ_40_, Aβ_42_, and Aβ_43_ can be efficiently measured by ELISA, and the existence of other longer peptides in vivo remains to be proven. Evidence as discussed in the main text indicates, however, that the ratio of short (Aβ_37_ + Aβ_38+_Aβ_40_) over long (Aβ_42_ + Aβ_43_) correlates linearly (*R*^2^ = 0.78, *p* < 0.0001) with the age of onset of familial Alzheimer’s disease in presenilin mutation carriers (Petit et al, 2022a).

The discovery that presenilins (PSEN) are responsible for the γ-secretase-mediated cleavage of the membrane carboxyterminal fragments (Fig. 1) of the amyloid precursor protein (APP) to the generation of the amyloid peptide (Aβ) provided a breakthrough in AD research (De Strooper et al, 1998). This finding unified the two major causes of inherited familial AD (FAD), i.e. mutations in the APP or the PSEN genes, in one molecular process: Aβ generation. It seemed also to provide a clear drug target for the field. At about the same time, it was demonstrated that presenilins/γ-secretases release the intracellular domain of Notch, which then travels to the nucleus and regulates Notch signaling (De Strooper et al, 1999; Struhl and Greenwald, 1999; Levitan and Greenwald, 1995). It transpired that Notch signaling was only one of the many signaling functions of the γ-secretases, contributing to the concept of regulated intramembrane proteolysis (Brown et al, 2000).

While these early breakthroughs created enthusiasm for the development of γ-secretase inhibitors (GSI), a deep understanding of the fundamental biology of this intriguing class of proteases was lacking. Ultimately this contributed to their failure in the clinic, largely due to unexpected adverse drug effects on the skin, the vascular and immune system, and cognition (Doody et al, 2013; Coric et al, 2015). A fundamental problem was the lack of selectivity of γ-secretase inhibitors that target all four γ-secretase isoforms. This blanket inhibition affected the proteolytic processing of hundreds of substrates in parallel (Hou et al, 2023), making it impossible to find an acceptable efficacy/side effect therapeutic window. Unfortunately, little was learned from the failed trials (De Strooper, 2014), and γ-secretases were largely deprioritized as drug targets for the industry in the context of AD. Interestingly, the capacity of GSIs to block Notch signaling (De Strooper et al, 1999) remained all this time of interest to companies in the context of cancer and chronic inflammation (McCaw et al, 2021; Christopoulos et al, 2021). Nirogacestat (Ogsiveo) recently became the first GSI to reach regulatory approval for rare desmoid tumors that are non-cancerous growths of the connective tissue (Gounder et al, 2023) (see Box 1).

Recent success with anti-amyloid antibodies (Sims et al, 2023; van Dyck et al, 2023) in AD have considerably changed the perspective on the potential of Aβ lowering as a possible therapy for this devastating disorder (Karran et al, 2011; Karran and De Strooper, 2022). However, various aspects of anti-amyloid immunotherapy may not make them the optimal therapeutic choice for primary prevention clinical studies. A possible approach to primary prevention might be the suppression of Aβ production so that the key Aβ plaque-seeding peptides are not produced in sufficient concentrations to initiate plaque formation. While inhibition of γ-secretase or of BACE1, the other protease that is needed to release Aβ from APP (Fig. 1), remain formal possibilities, both approaches suffer from the same dilemma: can a therapeutic window be established sparing the normal functions of both enzymes? To administer agents that already carry known adverse events to normal individuals lacks clinical equipoise. *Gamma-secretase allosteric stabilizers (GSAS)*, in contrast to γ-secretase inhibitors (GSI), theoretically allow normal physiological processing of the many substrates of the γ-secretases, while enhancing the processive cleavage of the Aβ peptide, favouring the generation of shorter forms as we will discuss below. The GSASs avoid the mechanism-based side effects that precluded the clinical development of GSIs and revert the production of all long Aβ peptides to shorter forms, in contrast to the earlier *γ-secretase modulators (GSM)* that were directed to Aβ_42_ lowering (Weggen et al, 2001; Luo and Li, 2022).

In the first part of this review, we will discuss our current understanding of γ-secretases and the effect of clinical mutations on their activity, resolving ongoing controversy in the field. We will stress how novel insights from cryo-EM structures (Yang et al, 2021) and a better understanding of function (Petit et al, 2022a; Szaruga et al, 2017) and cell biology (Sannerud et al, 2016) of the γ-secretases have allowed insights into the mode of action of GSASs. In the second part of the review, we will use these insights to explain the mode of action of GSASs and to discuss the roadblocks to further clinical development of GSAS-based precision medicine approaches for the prevention of AD.

Box 1 Complex-specific γ-secretase inhibitors (GSI) for the treatment of cancer and other diseases One of the major aims of the therapeutic development of GSIs in AD was to separate Notch from APP processing. The putative Notch sparing inhibitor avagacestat (Coric et al, 2015) made it to a phase-II clinical trial but was halted because of Notch and other side effects. Recent cryo-EM structures show that semagacestat and avagacestat bind close to the critical mixed β-sheath that stabilizes APP and Notch in the catalytic cleft of γ-secretase (Fig. 3) (Zhou et al, 2019; Yang et al, 2019). While it seems unlikely that GSIs will be reconsidered in the future for the treatment of AD because of these side effects, (selective) γ-secretase inhibition might be useful in other therapeutic areas (Jurisch-Yaksi et al, 2013), for instance, various cancers (Habets et al, 2019; Ranganathan et al, 2011) and hearing loss (Tona et al, 2014) among others, especially when Notch signaling is involved (Christopoulos et al, 2021; McCaw et al, 2021). Actually, nirogacestat (Ogsiveo) is the first GSI that reached regulatory approval for rare desmoid tumors (Gounder et al, 2023). While developing such Notch inhibitors, it is important to take into account that at least four different γ-secretase complexes exist (see main text: The discovery of γ-secretases) resulting from the four possible combinations of the constituting subunits PSEN1 or 2, APH1A or B, NCT, and PSENEN. These complexes have tissue-specific roles in Notch signaling as demonstrated by experiments with MRK560 (Churcher et al, 2006; Best et al, 2006), a GSI that suppresses T-cell Acute lymphoblastic leukemia (T-ALL) (Habets et al, 2019) without the classical Notch-related toxicity seen with broad spectrum GSIs in gut, skin or thymus. The compound MRK560 is selective for PSEN1 above PSEN2 γ-secretase, and PSEN2-γ-secretase is able to maintain the Notch physiological function in the gut and skin. The compound MRK560 was not further developed as a clinical candidate because of CYP2C9 inhibition liability (Zhao et al, 2015). Recent structural information explains the specificity of MRK560 at the molecular level (Guo et al, 2022; Serneels et al, 2023). It is thus possible to exploit the relatively small differences between the different complexes to make selective and more safe medication. In a recent comparison of 12 GSIs (Serneels et al, 2023), a few showed slight preference for APH1B over APH1A. New cryo-EM structures should explore the structural differences between APH1A or APH1B-containing complexes, which should help to generate specific drugs against APH1B-containing complexes; these are involved in amyloid plaque generation in an AD mouse model (Serneels et al, 2009).

## The discovery of γ-secretases

A pivotal study linked inherited forms of AD to missense mutations in S182, a gene of unknown function at that time (Sherrington et al, 1995). Soon thereafter, mutations in a second, homologous gene were identified (Rogaev et al, 1995). The genes were called *PSEN1* (abbreviation for Presenilin 1) and *PSEN2* to indicate their relationship with “presenile” early-onset AD (Rogaev et al, 1995). The function of PSENs was completely unknown, but similarities with other genes encoding multi-transmembrane domain proteins led to speculations about their potential roles in vesicle trafficking, ion channel activity, and Ca^2+^ signaling, among other possibilities (Sherrington et al, 1995). Some of these ideas are still prevalent in the field, although the first evidence for their function from work in *Caenorhabditis elegans* (Levitan and Greenwald, 1995) showed that the presenilin orthologue *SEL-12* is essential for Notch (Lin) signaling. The authors noted that *the remarkable conservation of SEL-12 and S182 does not provide any immediate indication of the function of S182 in the Alzheimer’s disease process*. Nevertheless, speculations about the role of Notch signaling in AD still remain. A more direct clue to their role in AD was the finding that presenilin missense mutations, when expressed in cell cultures, increased the generation of long, more aggregation-prone Aβ_42(43)_ peptides (Scheuner et al, 1996; Duff et al, 1996), but the underlying mechanism remained elusive. Finally, a simple knock-out experiment of *PSEN1* provided clarity showing that *PS1 (Presenilin 1) is involved in γ-secretase-mediated proteolytic cleavage of the C-terminal transmembrane fragments of APP after their generation by α- and β-secretase(s*) (De Strooper et al, 1998). Further work (Wolfe et al, 1999; Struhl and Greenwald, 1999; De Strooper et al, 1999) confirmed this conclusion and demonstrated “*a more direct role for PSEN1 as a regulatory or catalytic component of the protease(s) that cleave(s) Notch-1 and APP*” (De Strooper et al, 1999). The unequivocal proof that presenilin was indeed the catalytic protease cleaving Notch and APP was delivered when the aspartyl-protease transition state inhibitor L-685,458 was cross-linked to PSEN1 (Li et al, 2000).

**Figure 2:**
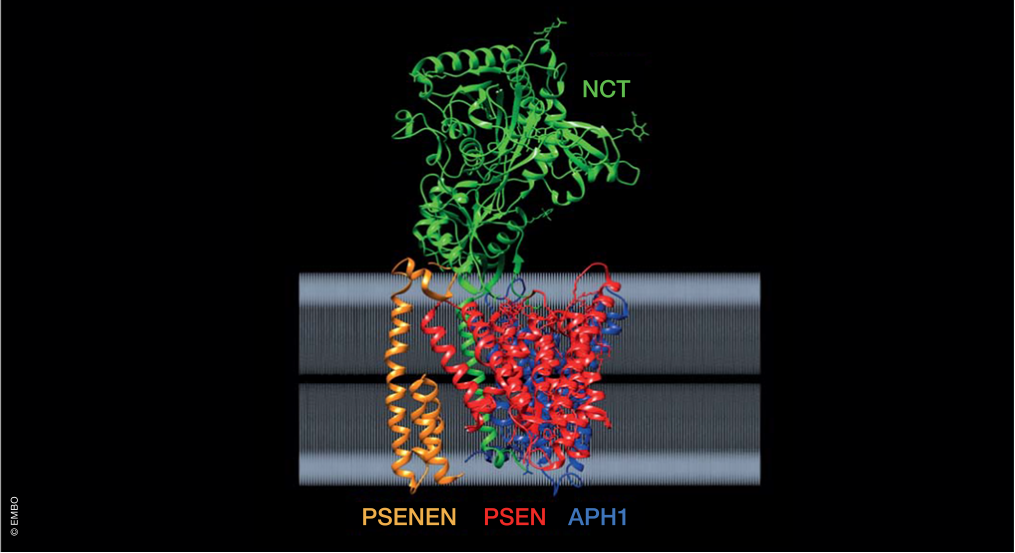
Gamma-secretase complex embedded in the cell membrane. The four subunits are indicated in green (Nicastrin - NCT), red (Presenilin - PSEN), blue (Anterior pharynx defective - APH1), and orange (Presenilin enhancer - PSENEN). The structural coordinates can be found at 10.2210/pdb7D8X/pdb (Yang et al, 2021). Analysis of the data was done using the chimera package (v1. 17.3) (Pettersen et al, 2004).

Presenilins only become active proteases when integrated into the γ-secretase complexes (Takasugi et al, 2003; Edbauer et al, 2003) (Fig. 2). These tetrameric complexes contain three other proteins, i.e., nicastrin (NCT or NCSTN), presenilin enhancer 2 (Pen-2 or PSENSEN), and anterior pharynx homolog 1 (APH-1) (reviewed in (De Strooper, 2003)). The assembly occurs stepwise with dimer formation in the endoplasmic reticulum and full assembly in the Golgi where the NCT subunit becomes fully glycosylated (Wouters et al, 2021). The first substrate of γ-secretase is the PSEN subunit itself, which becomes activated by auto-proteolytic cleavage (Thinakaran et al, 1996). As there are two PSEN genes and two APH1 genes, four different γ-secretase combinations exist (De Strooper, 2003). Additional complexity comes from alternative splicing, posttranslational modification, and transient association with other proteins and lipids, some of which regulate γ-secretase activity (Wong et al, 2020). The different complexes have rather divergent biological functions and therapeutic applications (see Box 1), as illustrated by the array of phenotypes caused by different genetic knockouts of the subunits. For instance, APH1A knock-out causes lethal Notch phenotypes during embryogenesis, while APH1B causes a mild behavioral phenotype in adulthood. PSEN1 and PSEN2 are present in different subcellular compartments. Complexes of PSEN1 are recycled between the cell surface and endosomes, while PSEN2 complexes are directed to late endosomes and lysosomes via binding to Adapter protein complex 1 (Sannerud et al, 2016). A more complete understanding of the heterogeneity in the structure, function, tissue distribution, and cellular compartmentalization of γ-secretases was not available during the preclinical and clinical development of the first GSIs (Doody et al, 2013; Coric et al, 2015).

## Missense mutations in presenilins cause Alzheimer’s disease

Presenilins PSEN1 and PSEN2 provide the catalytic cores to the different γ-secretases (De Strooper et al, 1998; Struhl and Greenwald, 1999; De Strooper et al, 1999; Wolfe et al, 1999; Li et al, 2000). Hundreds of different AD-causing mutations have been identified in the PSENs, but—remarkably enough—not in the other subunits of the complex (Rogaev et al, 1995; Sherrington et al, 1995). In fact, haploinsufficiency of PSEN and other subunits of the γ-secretase complex (Fig. 2) cause a skin disorder called hidradenitis suppurativa (Wang et al, 2010), a chronic skin disease characterized by painful, acne-like lesions and abscesses (Vellaichamy et al, 2021). There is no increased incidence of AD associated with this skin disease (Theut Riis et al, 2017; Garg and Strunk, 2017). In contrast to AD-causing mutations that affect only PSEN, the hidradenitis suppurativa—causing mutations occur mostly in NCTSN or PSENEN, the other subunits of the γ-secretase complex, and such mutations have never been found in AD patients. Deficient NOTCH signaling in hair follicles might explain the skin phenotypes, but this is far from settled (Zhang and Sisodia, 2015; Shi et al, 2023). It is also unclear why only skin is affected: deficient NOTCH signaling should affect many organs. The proteolytic function of γ-secretase certainly plays a role in the disease, as 9 out of 17 patients treated with the γ-secretase inhibitor niragacestat for desmoid tumor/aggressive fibromatosis developed hidradenitis suppurativa-like lesions (O’Sullivan Coyne et al, 2018). The lesions disappeared after halting the treatment. In summary, while these classical loss-of-function mutations cause hidradenitis suppurativa, the FAD mutations, in contrast, alter γ-secretase function without profoundly affecting its role in multiple signaling processes.

Heterozygous carriers of *PSEN1* mutations present with dominantly inherited early-onset AD (mean age of onset: 43.6 ± 7.2 years), and they display all cardinal features of sporadic AD with the full spectrum of Tau pathology, neuronal loss, and dementia. Some patients present additional atypical clinical phenotypes, such as myoclonus, seizures, pyramidal and extrapyramidal signs, and atypical neuropathology, such as cotton wool amyloid plaques (Ryan et al, 2016; Bergmans and De Strooper, 2010), but not hidradenitis suppurativa. Alzheimer’s disease caused by *PSEN2* mutations is extremely rare, and the age of onset is rather variable (45–88 years) (Sherrington et al, 1996). It is possible that less APP is processed via the PSEN2-γ-secretase complex, and that the production of pathological Aβ is quantitatively less important than in the *PSEN1* mutations.

While both *PSEN1* and *PSEN2* mutations are very rare causes of AD (Campion et al, 1999), their study has provided crucial insights into what constitutes pathological amyloid peptide (Aβ) generation (Szaruga et al, 2017; Veugelen et al, 2016; Kretner et al, 2016; Duff et al, 1996; Scheuner et al, 1996; Wagner et al, 2012), which has direct relevance to the understanding of sporadic AD (SAD), as we will discuss below. However, in our view, the genetic information was not, at the time, interpreted correctly by many in the field. The early-onset forms of AD caused by *PSEN* mutations were often referred to as being “aggressive” forms of the disease, and there was a belief that they led to overproduction of the Aβ_42_ peptide. In fact, nevertheless, there is only limited data currently supporting that FAD progresses more rapidly than SAD (Ryman et al, 2014), and in most cases, FAD *PSEN* mutations reduce, in some cases very significantly, the overall production of Aβ (Veugelen et al, 2016; Szaruga et al, 2015; Chávez-Gutiérrez et al, 2012; Shen and Kelleher, 2007; Xia et al, 2015).

Therefore, the consensus view is that the mutations cause a partial loss of PSEN function (Baumeister et al, 1997; De Strooper, 2007; Wolfe, 2007; Sun et al, 2017; Szaruga et al, 2015; Shen and Kelleher, 2007; Baumeister et al, 1997) and affect APP processing. The question of how this loss of function leads to AD remains, however, a contentious issue. The underlying conundrum is whether Aβ peptide and amyloid plaques are sufficient to trigger AD (“amyloid first”) (Selkoe and Hardy, 2016; Karran et al, 2011), or whether PSEN dysfunction itself is the cause of neuronal dyshomeostasis and neurodegeneration (“presenilin first”) (Shen and Kelleher, 2007). The latter hypothesis aligns with a school of thought that has criticized the amyloid hypothesis for AD and the idea that Aβ is the trigger of the disease. The “presenilin first” hypothesis is, however, largely based on experiments in conditional knock-out forebrain neurons or interneurons with total loss of PSEN1. This leads indeed to progressive neurodegeneration (Zhang et al, 2009; Xia et al, 2015; Watanabe et al, 2014), but in none of these conditions are amyloid plaques or neuronal tangles observed. Moreover, conditional knockouts of the other subunits of γ-secretase, i.e., *Ncstn* (Tabuchi et al, 2009) and *Aph1a* and *b* (Acx et al, 2017), mutations of which have not been associated with FAD, cause similar neurodegeneration. Membrane-bound fragments generated from App, Aplp1, Nrg1, Dcc, and other γ-secretase substrates (Acx et al, 2017) accumulate >10-fold in the targeted neurons. It is no surprise that this leads to severe disturbances of neuronal membrane functions, including synaptic transmission. Clearly, the “presenilin first” hypothesis fails to provide a consistent explanation for the neuropathology that characterizes FAD patients.

## Familial Alzheimer’s disease mutations in presenilins reveal the importance of long Aβ-seeds

Importantly, the loss-of-function mutations causing FAD are not null mutations and they always target the enzymatically active PSEN subunit. Gamma-secretases cleave their substrates in two steps (Quintero-Monzon et al, 2011). The first endoproteolytic cleavage occurs close to the cytoplasmic side of the transmembrane domain and is referred to as the ε-cleavage. This is the cleavage that releases the intracellular domains of APP, Notch, and other substrates and enables intracellular signaling (Jurisch-Yaksi et al, 2013) (Fig. 1). The remaining Aβ_48/49_ is further trimmed by consecutive γ-cleavages that progressively shorten the membrane-bound part of Aβ until it is released into the extracellular space (Takami et al, 2009). Every cleavage step requires the progressive unwinding of the transmembrane helix, the reengagement of the catalytic site, and the formation of a new enzyme-substrate complex. Recent structural studies show how APP is anchored in the complex via an induced mixed β-sheet structure formed between the carboxyterminal Aβ region of APP and two additional peptide-strands of PSEN. This positions the cleavage site of the APP substrate into the catalytic site and makes the first γ-cleavage of APP possible. A partial unwinding of the resulting Aβ_48/49_ is needed to expose the new cleavage site to the catalytic site of the protease (Zhou et al, 2019; Bhattarai et al, 2022). The carboxyterminal amino acids of Aβ_48/49_ are likely to form a new short β-strand to anchor itself in a new mixed β-sheet structure allowing the next cleavage to occur (Yang et al, 2019) (Fig. 1). Computer modeling shows that minimally three amino acids are required to stabilize this β-sheet, explaining why tripeptides are generated in the consecutive cleavage steps (Chen et al, 2023). It is assumed that the consecutive enzyme-substrate complexes become thermodynamically less and less stable as the Aβ sequence shortens (Szaruga et al, 2017) to Aβ_45_/Aβ_46_, Aβ_42_/Aβ_43_, and finally, to Aβ_40_, Aβ_37_, and Aβ_38_, which can all be detected in the extracellular medium (Fig. 1) (Funamoto et al, 2004; Takami et al, 2009; Sato et al, 2003; Quintero-Monzon et al, 2011; Bhattarai et al, 2022).

This model predicts that small alterations destabilizing the consecutive enzyme-substrate complexes will lead to premature release of long Aβ peptides: this has been demonstrated experimentally by increasing the incubation temperature of an in vitro γ-secretase assay and measuring the shift in the Aβ peptide length profile (Szaruga et al, 2017). Clinical mutations in *PSEN1* also destabilize the enzyme-substrate interactions, as shown by their increased temperature sensitivity (Szaruga et al, 2017). Thus, these mutations cause loss of γ-secretase function, which primarily affects the carboxypeptidase-like processing, and leads to the production of aggregation-prone long Aβ peptide. It is likely that the clinical *PSEN* mutations also affect the carboxypeptidase-mediated cleavages of transmembrane domains of other substrates (Weber et al, 2022), but only the Aβ peptide apparently has the property to form β-sheet fibrillar structures, explaining why PSEN mutations cause AD.

It should be stressed that most human carriers of *PSEN* mutations are heterozygous. Therefore, normal *PSEN1* and *PSEN2* alleles provide normal Notch and other signaling to the tissues, but also normal levels of Aβ peptides. The small amounts of longer Aβ peptide produced by the mutant *PSEN* allele, as discussed above, provide “seeds” that subsequently induce the aggregation of the Aβ generated from the normal γ-secretases into amyloid plaques (Veugelen et al, 2016). The more unstable the mutant complex, the less it contributes to the total amount of cellular Aβ produced, but the greater the proportion and concentration of the longer seeding peptides.

## Total short-versus-long Aβ peptide predicts disease onset in familial Alzheimer’s disease patients

A recent elegant study analyzing 25 different FAD mutations in *PSEN1* provides strong support for the “amyloid first” hypothesis. A linear correlation (*R*^2^ = 0.78, *p* < 0.0001) was established between age at onset of AD in the different carriers and the relative ratio of short-versus-long Aβ ((Aβ_37_ + Aβ_38_ + Aβ_40_)/(Aβ_43_ + Aβ_42_) (Petit et al, 2022a)). The data strongly corroborate the hypothesis that destabilizing effects of clinical mutations on the enzyme-substrate complex lead to the release of long (seeding) Aβ peptide (Szaruga et al, 2017), as illustrated by the increases in Aβ_43_ in several PSEN mutants. Importantly, an independent study confirmed a similar relationship between age at onset and long-versus-short Aβ in 162 PSEN1 FAD-causing variants (Schultz et al, 2023). It should be noted that robust assays for Aβ_>43_ (longer than 43 amino acids), which are likely generated by the most severe mutations, are currently lacking. A method to measure these longer Aβ peptides remains a priority for the field.

In conclusion, the study of gain and loss of function of the γ-secretases have yielded a plethora of insights into the normal and pathological functions of these intriguing enzymes in embryogenesis and in adulthood. These enzymes are essential for the homeostasis of tissues with the intestine, skin, immune system, and brain being particularly sensitive to alterations in their function. Clinical trials have demonstrated that a therapeutic window for γ-secretase inhibition is absent in AD. The evidence that PSEN mutations cause the generation of longer Aβ-peptides, however, suggests that an inverse mechanism, shifting the production to smaller peptides by stabilizing the APP-γ-secretase substrate-enzyme interaction could have a profound protective effect on the pathogenesis of AD. These studies also suggest that the focus of the field on Aβ_42_ production alone has been an oversimplification (see Box 2), as other long Aβ peptides are also produced and could, even when their total production is very low, have a catalytic effect on Aβ aggregation and amyloid plaque formation (Saito et al, 2011).

Box 2 Criticism of the “amyloid first” hypothesis The most serious criticism of the “amyloid first” hypothesis came from a comprehensive surview of the activities of 138 different *PSEN1* mutations tested in two cell-free reconstitution assays (Sun et al, 2017). The authors found that eight of the mutations (V96F, Y154N, K155_insFI, V261F, G394V, C410Y, L435F, and DeltaT440) abrogated endoproteolytic maturation of the complex and eliminated Aβ_40_ and Aβ_42_ production. Furthermore, a large majority of 104 out of 138 variants were producing lower levels of Aβ_40_ and Aβ_42_ peptides. As there was no significant correlation between the Aβ_42_/Aβ_40_ ratio produced by the different reconstituted γ-secretases and mean age at the onset of disease in patients carrying those mutations (Sun et al, 2017), the authors concluded that PSEN mutations do not cause AD via the production of long Aβ and that other mechanisms must play a role. However, when one extreme outlier (A260V) was removed from the data set a highly significant correlation between age of onset and Aβ ratio was restored (Tang and Kepp, 2018). In addition, as only two Aβ species were measured, Aβ_43_, which is increased in PSEN1 mutations L166R, L235P, G266S, R278T, L282R, and A431E (Petit et al, 2022a), was not accounted for in these experiments. Finally, detergents or liposomes, used for cell-free assays of γ-secretase, have a destabilizing effect on the enzyme-substrate interaction needed to process Aβ peptides. This could explain why the 8 most extreme variants (V96F, Y154N, K155_insFI, V261F, G394V, C410Y, L435F, and DeltaT440) did not show enzymatic activity in these assays. In support of this explanation: two of these mutants, when tested in cell culture, i.e., C410Y and L435F, produce small amounts of Aβ_43_ peptide and are thus active (Veugelen et al, 2016; Kretner et al, 2016). It is important to note that such very severe mutations like R278I (Saito et al, 2011), L435F (Veugelen et al, 2016; Kretner et al, 2016), and C410Y (Veugelen et al, 2016) affect not only the γ-cleavages but also decrease the ε-cleavage. In homozygote knock-in mice, this results in deficient Notch signaling and lethality during embryogenesis (Saito et al, 2011; Xia et al, 2015). However, they are not catalytically inert as they release small amounts of long Aβ in cell culture (Veugelen et al, 2016) and animals (Saito et al, 2011).

## Elucidation of the structure of γ-secretases facilitates new drug development

The elucidation of the atomic structures of γ-secretase (Lu et al, 2014) and γ-secretase bound to APP or Notch substrates (Zhou et al, 2019; Yang et al, 2019) have provided a molecular basis to understand the mechanism of these enzymes. The transmembrane domain of tetrameric γ-secretase consists of 20 transmembrane domain helices. This domain is organized in a horseshoe conformation (Bai et al, 2015b). The catalytic aspartates (Wolfe et al, 1999) D257 and D385 reside on the transmembrane domain (TM)6 and TM7, respectively, of PSEN, and they are located at the concave site of the horseshoe. The structure is topped by the large ectodomain of NCSTN that overlays the whole hydrophobic domain of γ-secretase on the extracellular surface (Fig. 1). To obtain structures from the in principle unstable enzyme-substrate complexes, Shi and colleagues (Yang et al, 2019; Zhou et al, 2019) introduced cross-linking cysteine mutations in both substrate and protease, and created a catalytically inactive enzyme by substituting aspartate D385 with Alanine. This, of course, blocks the autocatalytic activation of PSEN1. To mimic the effects of the autocatalytic PSEN activation, the PSEN1-NTF and -CTF were expressed separately.

The resulting cryo-EM structures (Zhou et al, 2019; Yang et al, 2019) are of remarkable quality, with resolutions of 2.8 and 2.7 Å, respectively. The novel structures reveal that several transmembrane domains of PSEN1 become reorganized and that unstructured parts of the enzyme become ordered when binding to its substrates (Yang et al, 2019; Zhou et al, 2019). A very interesting feature is the induction of a β-sheet structure between PSEN1 and its substrates that stabilizes the substrates in the catalytic cleft. This β-sheet consists of a β1 and a β2 strand at the end of transmembrane domains 6 and in the loop 2 domain of the PSEN protein, and an induced β3-strand in the Notch or APP substrates. Numerous other local rearrangements occur, for instance, the two catalytic aspartate residues, which are remote in the substrate-free structure become aligned and placed 6–7 Å away from the scissile peptide bond in Notch (Yang et al, 2019). Both APP-C83 and Notch-C100 substrates are accommodated in the same cut-through channel in the transmembrane part of γ-secretase (Zhou et al, 2019). Based on the steric constraints from the structure, it appears that substrates can only reach the catalytic channel via “lateral gating” between TM2 and TM6 of PSEN and threading their small extracellular fragment through the extended loop 1 between TM1 and TM2 (Yang et al, 2019).

In addition, the substrates undergo major conformational changes. A small N-terminal loop of Notch interacts with residues at the extracellular surface of PSEN and NCSTN. This loop domain is followed by a TM helix, which is on its C-terminal part unwound over one helical turn. This is followed by the already mentioned β3-strand. The unwinding of the helix exposes the scissile bonds of the substrate to the catalytic aspartates. The PAL motif in PSEN transmembrane domain 9 (Tomita et al, 2001), previously shown to be essential for catalytic activity (Wang et al, 2004), interacts with this unwounded part of the helix. Upon cleavage and release of the intracellular domain, the carboxyterminal end of the Aβ peptide is proposed to unwind over one helix and to reform again a new carboxyterminal β strand, initiating a new cycle of cleavage (Yang et al, 2019). This unwinding model, albeit not yet proven, could provide a mechanism for the consecutive cleavages of Aβ, which has tantalized the field for twenty years (Sato et al, 2003; Takami et al, 2009). It is logical to assume that the autocatalytic “presenilinase” cleavage (Thinakaran et al, 1996) of loop 2 also involves the induction of a third β-strand, provided by the PSEN-loop itself during assembly of the complex (Yang et al, 2019).

The different Aβ species are generated from APP by the progressive cutting of its transmembrane domain, which implies that it unwinds while in the γ-secretase catalytic cleft. The three helix-disrupting glycines in its transmembrane domain facilitate this process. Moreover, 11 out of the 18 amino acids in this domain are β-branched amino acids (e.g., Val, Ile) that might also contribute to the unwinding propensity of this domain (Yang et al, 2019). The consecutive cleavages result in a spectrum of Aβ peptides ranging from long (initially Aβ_49_, Aβ_48_, and then Aβ_46_, Aβ_45_, Aβ_43_, Aβ_42_) to short Aβ (Aβ_40_, Aβ_38_, and Aβ_37_) (Takami et al, 2009). At every step of this progressive shortening of the Aβ substrate a new enzyme-substrate complex is formed that is less stable, reflected in the stochastic release of the different peptides from the complex. Destabilization of the complex by clinical mutations (Szaruga et al, 2017) as explained, but also by alterations in the lipid composition in the membrane microenvironment of the enzyme (Winkler et al, 2012), or by external compounds (inverse modulators (Kukar et al, 2005)), increase the relative amount of long Aβ over short Aβ. Longer Aβ peptides are more prone to aggregate and to form oligomers and amyloid plaques, and they are therefore postulated here as the initial triggers of AD (Veugelen et al, 2016). The goal of γ-secretase allosteric modulation is to invert this process by stabilizing the complex so that shorter Aβ peptides are produced. As such, this should leave the initial ε-cleavage intact and, as a consequence, the signaling function of γ-secretase.

## Targeting γ-secretases for Alzheimer’s disease

The amyloid hypothesis (Selkoe and Hardy, 2016) is linear, quantitative, and neurocentric (De Strooper and Karran, 2016), leading to the assumption that simple lowering of Aβ (or Aβ_42_) is necessary and sufficient to reverse the cognitive deficits in patients. However, the amyloid hypothesis does not consider the complex, decade-long cellular disease process—involving glia and vascular cells- that underlies AD (De Strooper and Karran, 2016), and therefore the possibility that amyloid is necessary, but not sufficient to cause neurodegeneration. The cellular hypothesis proposes instead that the reaction of the brain cells to amyloid, determined by genetics and environment, leads to neurodegeneration and dementia. This hypothesis suggests many targets for disease modification in the cellular phase of the disease, downstream of the amyloid plaques. The cellular hypothesis, however, also implies that once neurodegeneration has been initiated, amyloid therapies will have only a limited effect on the clinical outcome. The recent clinical data from the phase-III trials with lecanemab (van Dyck et al, 2023) and donanemab (Sims et al, 2023) clearly show a separation of the placebo and treated arms indicating a genuine disease-modifying effect, but it is very unclear from the available data (Sims et al, 2023; van Dyck et al, 2023) whether the divergence is sustained or whether the curves become parallel, which is predicted by the cellular hypothesis. The cellular hypothesis also strongly suggests that primary prevention of oligomer and amyloid plaque generation before the occurrence of brain inflammation and damage should have a major impact on the incidence of AD.

An AD prevention therapy could be envisaged with a modest, and perhaps intermittent, dosing of an amyloid-clearing antibody, such that amyloid plaques or oligomers are removed early on, or, even better, prevented from developing. The three available drugs (aducanumab, lecanemab, and likely soon, donanemab) all cause, with varying incidence, brain vascular extravasation, microhaemorrhages, and rarely severe brain hemorrhages (Solopova et al, 2023; Castellani et al, 2023). This might be mediated by a vascular inflammatory response to amyloid angiopathy aggravated by anti-Aβ antibody. If this holds true, it would seem unlikely that a similar response would be seen in people who have low or no amyloid in their brains. Perhaps a more concerning aspect is the anti-amyloid antibody-mediated increase in brain atrophy, as evidenced by MRI (Alves et al, 2023). The time course of this atrophy is discordant with the time course of amyloid removal, which is evident in the study with bapineuzumab, that barely removed any amyloid from the brain (Salloway et al, 2014). The atrophy appears to relate to the presence of a plaque-binding antibody in the brain. In fact, it is not clear whether this atrophy is even a measure of neurodegeneration in this particular context. Nevertheless, this might present an unquantifiable risk in a primary amyloid prevention study with anti-amyloid antibodies and will require post-treatment monitoring.

Current agents are administered intravenously (although subcutaneous administration is being investigated). This somewhat burdensome feature is not optimal for subjects entering a primary prevention study for which multiple-year studies will be required, and does not compare favorably with the potential of a once-a-day tablet. Using these antibodies for primary prevention of AD seems, therefore, to be logistically very challenging.

The insights gained by studying the FAD mutations in PSEN are pivotal in this regard, as they show that the age of disease onset is determined by the relative ratio of long Aβ_≥42_ to short Aβ_≤40_. Allosteric modulators of γ-secretase shift Aβ peptide production from long to short peptides, and are, therefore, fundamentally acting inversely to the causal mutations in FAD. As the mutations bring the age of onset forward, such treatment should postpone (indefinitely) the onset of disease.

## The evolution of γ-secretase modulators to γ-secretase allosteric stabilizers

Gamma-secretase modulation was first demonstrated with non-steroidal anti-inflammatory drugs (NSAID), such as ibuprofen, indomethacine, and sulindac sulfide (Luo and Li, 2022; Weggen et al, 2001). At very high concentrations (25–300 µm) and independently from cyclooxygenase (COX) inhibition, these drugs lower significantly Aβ_42_, increase Aβ_38_, and do not affect Aβ_40_ in cell cultures and mice (Weggen et al, 2001). Tarenflurbil (R-flurbiprofen) was tested in a large phase-III trial, without success however (Green et al, 2009), likely because brain concentrations of the agent were insufficient to mediate a pharmacological effect (Karran and Hardy, 2014). In general, the first GSM generation lacked potency, and, besides some academic work (Saretz et al, 2021), this chemical space (Santiago et al, 2021; Mekala et al, 2020) has largely been abandoned.

A second generation of GSMs was developed based on the carboxyl-acid moiety of the NSAID scaffold. These compounds, similar to the first-generation GSMs, selectively affect Aβ_42_/Aβ_38_, while sparing Aβ_40_. Examples of the most potent compounds in these series are EVP-0015962 (Rogers et al, 2012), GSM2 (Kretner et al, 2011), and BIIB042 (Scannevin et al, 2016), all with EC_50_ values for Aβ_42_ lowering in the double-digit nM range. However, their drug-like properties were suboptimal and there are no reports of their effects in human studies (Mekala et al, 2020; Santiago et al, 2021).

These first and second generations of GSMs were screened empirically using cell-based assays and focusing on their Aβ_42_-lowering properties and their capacity to maintain the ε-cleavage of γ-secretases that is critical for intracellular signaling pathways. However, the mechanism of action and the precise target of these drugs remained unclear (Kounnas et al, 2010; Kukar et al, 2008; Kretner et al, 2011; Wanngren et al, 2012). The term “GSM” is used for any compound that decreases the Aβ_42_/Aβ_40_ ratio (Golde et al, 2013). This nomenclature, while useful at the outset to distinguish them from GSIs, fails to encompass our new understanding of γ-secretase enzymatic function.

The identification of the third generation of GSMs provided a major step forward and the first examples of GSASs. The prototype, introduced in 2006 by Eisai (Yu et al, 2014), targets an allosteric site in the PSEN subunit (Cai et al, 2017; Yang et al, 2021). The GSASs stabilize the enzyme-substrate complex to increase Aβ peptide processivity (Fig. 1), lowering the ratio of long Aβ_≥42_ to short Aβ_≤40_ peptides (Szaruga et al, 2017; Chávez-Gutiérrez et al, 2012; Petit et al, 2022b). In essence, they act in the inverse manner of FAD mutations, as discussed earlier. The properties of GSASs are summarized in Table 1. We propose to restrict the use of the name γ-secretase allosteric stabilizer (GSAS) strictly to this class of novel molecules (Table 1 and Table 2). Several molecules of this class are confirmed to bind to the same allosteric site on PSEN (Takeo et al, 2014; Ebke et al, 2011; Yang et al, 2021; Petit et al, 2022b).

**Table 1:** Characteristics of a gamma-secretase allosteric modulator (GAM).

**Pharmacological properties**			
Does not affect the epsilon cleavage of substrates			
Binds an allosteric site in gamma-secretase			
No effect on total Abeta			
Lowering effect on Abeta42 and 43			
No or moderate effects on Abeta 40			
Increasing effect on Abeta37/38			
No accumulation of APP-CTF			
Maintenance of Notch and other signaling pathways			
**Drug properties**			
Property	Good	Acceptable	Poor
TPSA	40–90	90–120	<20
		20–40	>120
H-Bond	<1	1–2	>2
Donor			
H-Bond	<4	4–8	>8
Acceptor			
MW	<360	360–500	>500
ClogP	<3	3–5	>5
ClogD	<2	2–4	>4
pKa	<8	8–10	>10
Kp,uu (brain)	>0.3		

The essential pharmacological properties are summarized and defined as whether a compound can be considered an allosteric modulator of γ-secretase. All criteria should be checked and fulfilled. The drug properties are important for any drug targeted to the central nervous system.

TPSA, topical polar surface area in Å2; H-Bond, hydrogen-bond; MW, molecular weight; ClogP, calculated logarithm of partition coefficient between n-octanol and water; ClogD, calculated logarithm of distribution coefficient between n-octanol and water at pH7.4; pKa, -logarithm acid dissociation constant; Kp,uu, ratio of unbound drug in brain versus plasma.

**Table 2:** Examples of ʏ-secretase allosteric modulators.

GAM or GSM	Chemical structure	Clinical trials	IC50*	Effects on Abeta	Notes and ref
E2012	aryl-imidazole	phase-I stop		↓ Aβ40, Aβ42 ↑ Aβ37, Aβ38	lenticular opacities and cholesterol metabolism problem in rat (EISAI)^1^
E2212	phenylimidazole	phase-I	9.0 nm	↓ Aβ40, Aβ42	EISAI^2^
NGP555, (compound 4)	thiazole	phase Ia + Ib	9 nm	lowers Aβ42 and plaques in rodents	Docked in ʏ-secretase complex (NeuroGenetic Pharmaceuticals)^3^
PF-06648671	pyridopyrazine-1,6-dione	Three phase-I 120 persons		↓ Aβ40, Aβ42, ↑Aβ37,Aβ38 →total Aβ (human csf)	Discontinued (Pfizer) ^4^
BMS-932481 (compound 12)	pyrimidine based	phase-I acute and chronic dosing/	6 nm	↓ Aβ40, Aβ42, ↑Aβ37, Aβ38 →total Aβ (human csf)	liver problems, discontinued (BMS)^5^
RO7101556	triazolo-azepine	no clinical data reported	10–20 mg/kg (mouse)	↓ Aβ40, Aβ42, ↑ Aβ38	Hoffmann-Laroche^6^
RG6289	n.a.	Phase I 180 persons	n.a.	↓ Aβ42	Hoffmann-Laroche^9^
FRM-024 (compound 41)	phenyl-oxadiazine	no clinical data reported	9 nm	↓ Aβ42 ↑Aβ37	Forum Pharmaceuticals^7^
UCSD-776,890 (compound 1)	heterocyclic, imidazole. pyridazine	no clinical data reported	5 nM	↓ Aβ40, Aβ42, ↑Aβ37, Aβ38	University of California and Massachusetts General Hospital^8^

The GSASs that have been tested in patients are indicated. The information was derived from the following references: ^1^(Nakano-Ito et al, 2014); ^2^(Yu et al, 2014); ^3^(Kounnas et al, 2017); ^4^(Ahn et al, 2020); ^5^(Soares et al, 2016); ^6^(Ratni et al, 2020); ^7^(Bursavich et al, 2021); ^8^(Rynearson et al, 2021) and ^9^https://www.alzforum.org/news/conference-coverage/second-generation-g-secretase-modulator-heads-phase-2.

The prototypes E2012 and E2212 have been tested in human phase-I trials demonstrating target engagement (Table 1). Trialing of E2012 was halted because of non-mechanism-based toxicity (cataracts in rats). Although E2122 was initially proposed as a safer alternative (Yu et al, 2014), undisclosed reasons have also halted the clinical development of E2122. A large variety of similar heterocyclic phenylimidazole compounds have been generated, but clinical progress was limited mostly because of non-mechanism-based liver toxicity (Mekala et al, 2020). Nevertheless, as indicated in Table 2, these are potent compounds (low single-digit nanomolar range in cell culture experiments), which decrease plaque formation and improve behavior in preclinical models of AD (for example (Kounnas et al, 2010; Rynearson et al, 2021)), while some show little preclinical toxicity as well. The compound NGP555 (Kounnas et al, 2019) demonstrated moderate pharmacodynamic effects in phase-I studies with a significant increase in Aβ_38_ but only a tendency to lower Aβ_42_. The compound BMS-932,481 (Soares et al, 2016) had the desired effects on Aβ: decreased Aβ_42_ and Aβ_40_, and increased Aβ_37_ and Aβ_38_ without effecting total Aβ in humans. However, liver toxicity limited the dose, and further clinical development has stopped. From the published work, compound PF-06648671 (Ahn et al, 2020) appears promising. Three phase-I clinical trials involving in total of 120 patients exposed to single and multiple-ascending doses have been reported (Ahn et al, 2020). Overall, an acceptable safety profile and a reduction in Aβ_42_ and Aβ_40_, with concomitant increases in Aβ_37_ and Aβ_38_ in the cerebrospinal fluid (CSF), without any effects on overall Aβ production were observed. This compound, at face value, has an appropriate GSAS profile (Table 1). In 2018, the company that had generated this compound announced that it was terminating its internal research efforts in AD (https://www.reuters.com/article/us-pfizer-alzheimers-idUSKBN1EW0TNf), and the fate of this compound series is unclear. Recently, phase-I data on RG6289, a novel GSAS, were presented (CTAD 2023), demonstrating robust reductions in CSF Aβ_42_ and Aβ_40_ levels with elevations in Aβ_38_ and Aβ_37_ (Table 2).

In conclusion, the available chemical, preclinical, and patient data suggest that it is possible to generate effective GSASs that have the desired effects in the human brain with likely a reasonable safety profile, although currently data on chronic dosing are missing. For many of these compounds, information on the criteria outlined in Table 1 remains only partially available, most crucially regarding the maintenance of the signaling function of the ʏ-secretases (Hou et al, 2023).

## The mechanism of action of γ-secretase allosteric stabilizers

In addition to having good drug-like properties, a critical feature of a GSAS is to leave the initial ε-cleavage by γ-secretases intact (Lessard et al, 2015, 2020; Page et al, 2008; Golde et al, 2013). This maintains the signaling function of γ-secretases and avoids the unwanted accumulation of unprocessed APP-CTF fragments (Im et al, 2023) in the membrane (Table 1). The GSASs should not alter the total Aβ generated, a potential advantage given the possible physiological roles of Aβ in synaptic function (Abramov et al, 2009; Fogel et al, 2014). As GSASs affect the processivity of the γ-secretase, strong lowering of Aβ_42_ and Aβ_43_, moderate or no lowering of Aβ_40_, and increases in Aβ_38_ and Aβ_37_ should be demonstrated.

The imidazole-based allosteric GSMIII increases Aβ cleavage processivity, but at high concentrations (3 µM) it increases also the initial ε-cleavage. While this—as the authors noted (Petit et al, 2022b)—might be an additional advantage considering the potential pathogenicity of APP-CTF (Im et al, 2023), it is unclear whether and how this would increase other signaling pathways in which γ-secretase is involved. It is likely better to optimize GSASs along the criteria set out in Table 1. As already mentioned above, GSMIII and probably other GSMs can cause shifting of the production line of Aβ peptides. The GSMIII favors Aβ_42_ and Aβ_38_ as end products (Petit et al, 2022b). The acidic GSMI also increases mainly Aβ_42_ to Aβ_38_ turnover. Its binding mode (and allosteric pocket) is different from the imidazole GSMIII binding pocket (Petit et al, 2022b). Clearly, a variety of agents produce a range of pharmacological profiles of γ-secretase function.

**Figure 3:**
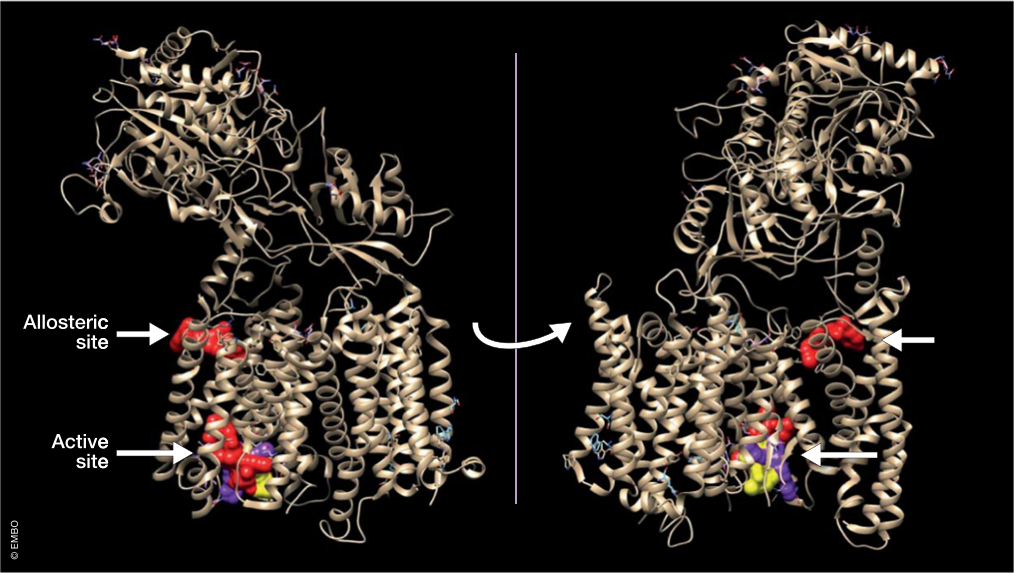
Binding sites of allosteric and orthosteric γ-secretase modulators and inhibitors. The ribbon structure of the γ-secretase complex is displayed and turned to show the binding sites of the allosteric modulator E2012 (red) and the inhibitors L625,458 (red), Semagacestat (yellow), and Avagacestat (magenta). The three inhibitors bind closely to each other at the catalytic site of the protease, while the modulator occupies the allosteric site in the complex. The structural coordinates can be found at 10.2210/pdbxxxx/pdb using PDB6LR4, 7D8X, and 6LGQ as pdb coordinates (Yang et al, 2021). Analysis of the data was done using the chimera package (v1. 17.3) (Pettersen et al, 2004).

The discovery of GSMs and GSASs was challenging from the compound screening and medicinal chemistry perspectives. The target does not lend itself to a facile biochemical screen, such as the one that was used successfully with BACE1 inhibitors. The measurement of compound activity was also complex: a range of Aβ proteoforms has to be measured to ensure the correct profile of activity is achieved. However, while challenging, by the time this research area was being deprioritized in many pharmaceutical companies, sufficient progress had been made to give confidence that clinically developable compounds might be found. The availability of highly resolved γ-secretase structures (Fig. 3) (Yang et al, 2021) opens the path to computer modeling, in silico screening, and rational drug design approaches (Ioppolo et al, 2021). The binding site of the prototype imidazole-based GSAS E2012 (Yang et al, 2021; Cai et al, 2017) is at the interface between PSEN1 and NCSTN on the extracellular side of the complex and partially covers the substrate-binding tunnel (Fig. 3). This overlaps with the binding mode of the amino-terminal portion of the APP substrate (Petit et al, 2022b; Yang et al, 2021). The compound NGP555 was computationally docked into the same site (Kounnas et al, 2017) based on an earlier, less well-resolved structure. Further exciting work (Petit et al, 2022b) using site-directed mutagenesis and in silico modeling demonstrated that another imidazole-based GSAS also potentially binds to the same site. Based on further functional work, the authors proposed a dual mode of action for this GSMIII: allosteric facilitation of the transition state and stabilization of the E-S complex by increased hydrophobic interactions of the shorter Aβ substrates with the complex (Petit et al, 2022b). The narrow structural and functional link between the substrate-binding site and the allosteric modulation site (Yang et al, 2021; Petit et al, 2022b) suggests that substrates and GSASs influence each other’s binding, stressing the importance of studying the molecular dynamics of the complex.

## Challenges to γ-secretase allosteric stabilizers as a precision medicine approach to Alzheimer’s disease prevention

Demonstrating AD prevention, defined in this context as the prevention of amyloid deposition, is a time-consuming and expensive clinical endeavor, no matter the therapeutic modality employed. Normally, one would expect to demonstrate clinical efficacy in AD before moving to secondary (treating amyloid-positive subjects) and primary prevention. For GSASs, however, it seems unlikely that they would show efficacy in those patients where significant levels of amyloid are already deposited. From the verubecestat phase-III studies, it is known that profound suppression of all Aβ species did not result in a robust reduction in plaques over 18 months (Egan et al, 2019). Most GSMs tested in preclinical studies in transgenic mice harboring FAD mutations that cause amyloid deposition have demonstrated prevention of amyloid deposition, i.e., tested compounds were administered prior to plaque deposition (Rogers et al, 2012; Van Broeck et al, 2011; Imbimbo et al, 2009; Scannevin et al, 2016; Brendel et al, 2015) rather than in mice with pre-existing amyloid pathology (Murakami et al, 2016) (Egan et al, 2019). Therefore, clinical development would have to focus on primary prevention: how could this be envisioned?

For the purposes of this discussion, let us assume a GSAS has demonstrated the appropriate effect on Aβ peptide production in preclinical cell and human APP transgenic mouse models; further, that there is a sufficient efficacy:safety margin based on toxicological findings.

Firstly, there is the critical importance of sustaining clinical equipoise. For whom would this approach be appropriate, and who would be motivated to participate in clinical studies? Clearly, PSEN mutation carriers would likely be enthusiastic participants, in theory. But with the advent of anti-amyloid antibodies (lecanemab and donanemab), such patients have alternative options. Indeed, lecanemab is currently undergoing clinical testing under the aegis of the DIAN-TU consortium (ClinicalTrials.gov Identifier: NCT05269394) in mutation carriers with separate symptomatic and non-symptomatic cohorts and in combination with an anti-tau antibody. Another concern is that some PSEN mutations confer resistance to GSMs and possibly GSASs, likely because they affect the binding pocket (Lessard et al, 2015; Page et al, 2008).

Another appropriate cohort may be amyloid-negative, ApoE4 carriers who are at higher risk of developing AD, but for whom disease symptomatology is not an inevitable consequence. The burden of receiving biweekly or monthly intravenous infusions (or, in the future, subcutaneous injections) of an anti-amyloid antibody for several years in a preventative study might lead to a significant drop-out rate, as compared, say, to a daily tablet. In a primary prevention study, however, it seems likely that a low antibody dose, perhaps administered on a much longer time interval, would be sufficient to keep amyloid levels very low, thereby diminishing the participant burden. For example, this is the approach being taken in the AHEAD 3-45 studies with lecanemab (Rafii et al, 2023). Besides some of the reservations discussed above regarding potential issues when using the existing antibodies in prevention trials, being able to take a daily tablet would likely be significantly preferred to a regular subcutaneous injection over a duration of many years.

A third potential path to the clinic is a secondary intervention trial where patients, after treatment with one of the amyloid plaque-clearing antibodies and after becoming amyloid PET-negative, are transferred to a trial with GSASs to prevent re-accumulation of the amyloid plaques. The Donanemab phase-III trial shows a path forward to such a trial, as the antibody treatment was stopped once the patients became amyloid PET scan-negative (Sims et al, 2023). While BACE1 inhibitors could be considered for such a combination therapy as well, there are serious mechanism-based side effects that might limit the dose used for chronic BACE1 inhibition, as discussed above. Furthermore, GSASs have the distinct advantages of not changing the level of the Aβ species in contrast to BACE1 inhibition, to avoid the mechanistic side effects associated with pure inhibition of proteases, and finally, to do precisely what is crucial: lowering all long Aβ species.

A GSAS clinical study would first proceed via a traditional single ascending dose (SAD) and multiple-ascending dose (MAD) approach to assess safety, pharmacokinetics, and pharmacodynamic effects in small numbers of healthy controls. Cerebrospinal fluid sampling would be taken to establish that GSAS therapy alters the ratio of Aβ proteoforms appropriately. Cells transfected with PSEN mutants secrete Aβ proteoforms and the short/long Aβ ratios changes from the wild type correlate well with the age of disease onset in patients harboring each mutation (Petit et al, 2022a). From this analysis, it was demonstrated that a 25% change in the Aβ (Aβ_37_ + Aβ_38_ + Aβ_40_)/Aβ_42_ + Aβ_43_) ratio in favor of the longer proteoforms compared to the wild type correlated with a 13-year lowering in the average age of disease onset. It is not unreasonable, therefore, to expect that a similar change in Aβ proteoform ratio in favor of the shorter forms would result in a 13-year delay in disease onset of sporadic AD—a 20-year delay would effectively prevent AD. This 25% change could be set as the minimum pharmacodynamic effect evidenced in a MAD study that would provide confidence to embark on a phase-II dose-ranging study. However, if no adverse side effects were experienced, the phase-I MAD study could increase the drug exposure levels until the pharmacodynamic effect of increasing the ratio of shorter/longer Aβ proteoforms reached an asymptote.

A phase-II study should seek to demonstrate the safety of the therapeutic approach, evidence of efficacy or target engagement, and provide sufficient data to be able to either accept the null hypothesis or power a phase-III study in terms of therapeutic dose, group size, clinical trial duration, and primary outcome measure. A critical aspect of a GSAS phase-II study is the participant brain amyloid status at baseline. The GSAS therapeutic hypothesis posits that the initial amyloid seeding events will be prevented or significantly delayed. What is not known is whether GSAS therapy will, by suppressing the synthesis of the most amyloidogenic species, prevent further deposition in a brain with a low level of brain amyloid or even facilitate plaque resolution over time. In this context, “amyloid negativity” needs to be carefully defined (see Box 3).

By the time a phase-III study could be run, the regulatory environment may have changed such that prevention of amyloid accrual would be a surrogate endpoint. If cognitive and functional endpoints are required by regulatory bodies, an expensive trial will be needed. From longitudinal data on amyloid accrual (Sperling et al, 2023; LaPoint et al, 2022; Villemagne et al, 2013; van der Kall et al, 2021), an amyloid primary prevention study would have to be of many years’ duration with large group sizes even using the sensitive Preclinical Alzheimer Cognitive Composite (PACC) 5 assessment scale. An issue yet to be resolved is whether the magnitude of deflection of cognitive decline in very early disease will be deemed as being clinically meaningful by regulators and healthcare providers more generally (Insel et al, 2019).

Nevertheless, we should take lessons from other areas of medicine development. Large-scale clinical trials have been cornerstones for trialing in cardiovascular medicine and have delivered the evidence base for treatments currently saving many years of life throughout the population. A large-scale cardiovascular study enrolls typically 5000–20,000 patients and might take 7–10 years to complete (Packer and Pitt, 2018). Of note, approval for the first statin for human use in 1987 was based on studies showing that it lowers plasma LDL and is well-tolerated. There was no evidence that it could prevent heart attacks. This evidence was delivered only in 1994 with a second-generation statin in a large follow-up study (Goldstein and Brown, 2015).

The precision medicine approach that GSASs represent is theoretically very attractive, while the clinical trial execution would be very challenging. A therapeutic that will be administered to “at risk” individuals has to be very safe, especially as the risk, in this case, amyloid deposition, might lead to clinical symptoms only many years later. Longer-term clinical trials have not taken place with GSASs, and thus their adverse event profile and efficacy remain unknown. It is mandatory that further preclinical and clinical research is performed to explore this highly interesting avenue toward preventive therapy (see Box 4).

Box 3 Assessing amyloid negativity in a γ-secretase allosteric stabilizer (GSAS) trial Various cut-offs for amyloid negativity have been assigned for different amyloid PET ligands using various methodologies (La Joie et al, 2019; Landau et al, 2014; Jack et al, 2017a,b). However, brains below that cut-off will be a mixture of genuinely amyloid-negative brains and brains with low levels of amyloid. The Centiloid project has provided guidance on how to convert the amyloid plaques signals obtained with different amyloid PET ligands into a common, 100-point scale (Klunk et al, 2015). Two studies that examined the issue (Salvadó et al, 2019; Farrell et al, 2021) determined that an appropriate Centiloid (CL) cut-off below which brains could be confidently assigned as being without amyloid deposits was 12 CL or <15–18 CL, respectively. Trial participant inclusion could be initiated by genotyping cognitively normal subjects between the ages of 60–67 years and selecting ApoE4 positives (Burnham et al, 2020; Bilgel et al, 2016; Jansen et al, 2022). Currently, a range of plasma biomarkers are being explored that correlate well with brain amyloid status (Jack et al, 2023). Recently, also an elevation in plasma pTau231 has shown utility in identifying amyloid-positive subjects, and plasma pTau231 levels with *Z*-scores = 2 correlated with an amyloid level of 26 CLs (Milà-Alomà et al, 2022). Thus, including participants with pTau231 *Z-* scores >1 < 2 could be employed as a secondary filter. The amyloid PET ligand with the best sensitivity and signal-to-noise characteristics is ^18^F-NAV4694 (Krishnadas et al, 2022; Rowe et al, 2016), which can detect very early amyloid deposition. Trial participants would have their amyloid PET status confirmed using 18F-NAV4694, with patients with >30 Centiloids being excluded. Based on the ApoE4-positive control subjects with baseline amyloid levels <12 CL, a group size of 143 subjects would be required to demonstrate a 75% reduction of amyloid accrual in a study of 2 years’ duration^1^.

Box 4 Questions and future directions Gamma-secretase research peaked in 2012 with 765 publications, but has drastically slowed down over the last years. It seems crucial that this work gets again the attention needed as understanding the role of these complexes in Alzheimer’s disease and Cancer could provide therapeutics in two major areas of medical need. Important areas of research are:

1. The (patho-)biological function of the small peptides generated by γ-secretases from its many substrates. Accurate measurement methods are available only for Aβ, with the exception of Aβ_≥43_ forms. Sensitive assays of the whole spectrum of Aβ peptides and an understanding of their physiological roles are needed. Additionally, more work on the hundreds of γ-secretase substrates and their metabolites are required (Hou et al, 2023).
2. A major question is how γ-secretase processivity is affected by endogenous or exogenous stimuli. The membrane localization of γ-secretase determines its activity (Thathiah et al, 2013). In neurons, electrophysiological activity can modulate long versus short Aβ ratio (Dolev et al, 2013). The innate immunity protein IFITM3 regulates its activity (Hur et al, 2020). The potential relevance of all these effects remains, however, largely unknown.
3. While an understanding of the heterogeneity of the γ-secretases exists (Box 1), there is a lack of insight into the biological relevance of different γ-secretase complexes in health and disease.
4. Patients with familial Alzheimer’s disease have the classical signs of Alzheimer’s disease, but many present with additional phenotypes. Understanding whether other substrates are affected by the presenilin mutations will help us to understand better the normal functions of γ-secretases and will help to develop better medication for these patients.
5. Dynamic studies of the γ-secretase complexes and how allosteric modulation affects the enzymatic activity of those enzymes are needed. The cryo-EM structures have provided deep insight into the previously elusive intramembrane proteolysis of APP and Notch. Nevertheless, these structures provide only snapshots of a complex multi-step process. Intermediary structures must exist, for instance, when the substrate docks to the complex. Cryo-EM approaches might elucidate such intermediary structures (Bai et al, 2015a), but kinetic modeling combined with informative site-directed mutagenesis will be important to fully unravel the function of these remarkable structures (Bhattarai et al, 2022; Chen et al, 2023; Petit et al, 2022b).
6. Alzheimer’s disease prevention would have a profound effect on global societal well-being. Therapeutic agents are now becoming available that have the potential to realize this ambition. In the history of medicine, it is rare that the first available agents are the best. With this in mind, it is important that the field continues to treat existing agents as a benchmark from which to improve, rather than as being a final answer.

## Conclusion

In many ways, the history of γ-secretases seems a repetition of other histories in medicine. By substituting long Aβ for LDL, we could just repeat what Noble laureates Brown and Goldstein wrote 8 years ago reviewing a century of cholesterol and coronaries: from plaques to genes to statins (Goldstein and Brown, 2015):

Few, if any, chronic diseases have been subjected to such intensive scrutiny, and rarely has the cause and the approach to prevention been documented so convincingly. It does not seem an exaggeration to state that targeted application of an LDL-lowering regimen may eventually curtail one of the major killers of the last century. The key questions for the 21st century are who to target and when. Ideally, LDL-lowering therapy should be initiated before atherosclerotic plaques develop or at least before they develop their most threatening features.

We know that AD presents an increasingly important healthcare problem, and the advent of the first disease-modifying drugs represents a towering breakthrough: the combined effort of thousands of basic and clinical scientists in academia and industry, and most importantly, the willingness of patients to enter clinical studies. However, the major growth in AD patients worldwide is going to be in low and middle-income countries (Martin Prince et al, 2015). The healthcare infrastructure of these nations is not yet sufficiently mature for large-scale deployment of antibody therapeutics. In summary, it seems sensible to have more, rather than fewer, therapeutic options available.

## Footnotes

1 The estimated sample size required for a primary prevention trial in Alzheimer’s Disease (AD) was determined by selecting healthy control, APOE4-positive carrier subjects with baseline centiloid values below 12 from the ADNI database. These subjects had baseline centiloid values of 0.77 ± 6.76 (mean ± std) and exhibited a 2.85 ± 6.45 (mean ± std) increase in centiloid over a 2-year period. A power analysis of this data suggests that a primary prevention trial to detect a 75% slowing of amyloid accrual would require 143 subjects per group for a 2-year trial using a two-group *t*-test with 80% power and a 0.05 two-sided significance level, calculated using the nQuery 8.1.2.0 software.
